# Short- and Long-Read Sequencing Reveals the Presence and Evolution of an IncF Plasmid Harboring *bla*_CTX-M-15_ and *bla*_CTX-M-27_ Genes in Escherichia coli ST131

**DOI:** 10.1128/spectrum.00356-23

**Published:** 2023-07-19

**Authors:** Rory Cave, Mary M. Ter-Stepanyan, Hermine V. Mkrtchyan

**Affiliations:** a School of Biomedical Sciences, University of West London, London, United Kingdom; b Yerevan State Medical University after M. Heratsi, Faculty of Public Health, Department of Epidemiology, Yerevan, Republic of Armenia; c Research Center of Maternal and Child Health Protection, Yerevan, Armenia; University Paris-Saclay, AP-HP Hôpital Antoine Béclère, Service de Microbiologie, Institute for Integrative Biology of the Cell (I2BC), CEA, CNRS

**Keywords:** *E. coli* ST131, ESBL UPEC, hybrid assembly, long-read sequencing, short-read sequencing, whole-genome sequencing

## Abstract

Escherichia coli sequence type 131 (ST131) has contributed to the spread of extended-spectrum beta-lactamase (ESBL) and has emerged as the dominant cause of hospital- and community-acquired urinary tract infections. Here, we report for the first time an in-depth analysis of whole-genome sequencing (WGS) of 4 ESBL-producing E. coli ST131 isolates recovered from patients in two hospitals in Armenia using Illumina short-read sequencing for accurate base calling to determine their genotype and to infer their phylogeny and using Oxford Nanopore Technologies long-read sequencing to resolve plasmid and chromosomal genetic elements. Genotypically, the four Armenian isolates were identified as part of the *H*30Rx/clade C2 (*n* = 2) and *H*41/clade A (*n* = 2) lineages and were phylogenetically closely related to isolates from the European Nucleotide Archive (ENA) database previously recovered from patients in the United States, Australia, and New Zealand. The Armenian isolates recovered in this study had chromosomal integration of the *bla*_CTX-M-15_ gene in the *H*30Rx isolates and a high number of virulence genes found in the *H*41 isolates associated with the carriage of a rare genomic island (in the context of E. coli ST131) containing the S fimbrial, salmochelin siderophore, and microcin H47 virulence genes. Furthermore, our data show the evolution of the IncF[2:A2:B20] plasmid harboring both *bla_CTX_*_-M-15_ and *bla*_CTX-M-27_ genes, derived from the recombination of genes from an IncF[F2:A−:B−] *bla*_CTX-M-15_-associated plasmid into the IncF[F1:A2:B20] *bla*_CTX-M-27_-associated plasmid backbone seen in two genetically closely related *H*41 Armenian isolates.

**IMPORTANCE** Combining short and long reads from whole-genome sequencing analysis provided a genetic context for uncommon genes of clinical importance to better understand transmission and evolutionary features of ESBL-producing uropathogenic E. coli (UPEC) ST131 isolates recovered in Armenia. Using hybrid genome assembly in countries lacking genomic surveillance studies can inform us about new lineages not seen in other countries with genes encoding high virulence and antibiotic resistance harbored on mobile genetic elements.

## INTRODUCTION

Escherichia coli sequence type 131 (ST131) is the most dominant global lineage of E. coli to cause community and hospital-acquired urinary tract infections (UTI) (1, 2). The high prevalence of ST131 isolates resistant to fluoroquinolones and beta-lactams and their association with the carriage and dissemination of the extended-spectrum-beta-lactamase (ESBL) antibiotic resistance gene *bla*_CTX-M-15_ have been documented (3–8). Based on their phylogenetic relationship, ST131 isolates have been divided into three main clades: clade A (predominantly represented by serotype O16:H5 *fimH41* allele isolates), clade B (predominantly represented by serotype O25b:H4 *fimH22* isolates), and clade C (predominantly represented by serotype O25b:H4 *fimH30* isolates) (9). Furthermore, clade C is further divided into three subclades: C0 (*H*30 lineage, which includes fluoroquinolone-sensitive isolates), C1 (*H*30R lineage, including fluoroquinolone-resistant isolates), and C2 (*H*30Rx lineage, including fluoroquinolone-resistant isolates with a G723A single nucleotide polymorphism [SNP] in the putative allantoin permease gene *ybbW* and carrying the *bla*_CTX-M-15_ ESBL gene) (10–12). The carriage of the *bla*_CTX-M_ ESBL gene in ST131 isolates is linked to a specific incompatibility group F (IncF) conjugational plasmid type that is also associated with a particular clade C lineage. The most common of these plasmid types is Inc[F1:A2:B20], which is associated with the ST131 C1 lineage and carriage of the *bla*_CTX-M-27_ gene, as well as Inc[F2:A1:B−], which is associated with the ST131 C2 lineage and carriage of the *bla*_CTX-M-15_ gene (9, 12, 13).

Studies reporting on the transmission and the genetic makeup of E. coli ST131 are well documented globally, including those that have used a combination of standard molecular techniques such as PCR and whole-genome sequencing analysis using short-read sequencing platforms with high throughput and accurate base calling to infer bacterial genotype and phenotype (11, 14–16). Furthermore, advances in long-read sequencing have enabled us to resolve repetitive elements so that we can identify the structure and positioning of mobile genetic elements (MGE) in chromosomes and plasmids, such as antibiotic resistance and virulence genes (17–20). However, such genomic studies reporting whole-genome analysis of the E. coli ST131 lineage recovered in health care settings (including patients) in Armenia are absent. In this study, we report for the first time the whole-genome sequencing and analysis of the ST131 lineage of ESBL uropathogenic E. coli (UPEC) ST131 isolates recovered from patients’ urine specimens in two hospitals in Armenia. To conduct the genomic analysis on Armenian ESBL-producing UPEC ST131, we used both Illumina and Oxford Nanopore Technologies (ONT) sequencing technologies to provide an in-depth genomic analysis identifying novel or rare genetic features within the Armenian isolates.

## RESULTS

### Isolates and antibiotic susceptibility testing.

Four of 12 ESBL-producing UPEC isolates were identified as ST131 isolates (see Table S1 in the supplemental material). The four isolates identified as ST131 were received from medical microbiology laboratories (they were recovered from urine specimens from patients) of two hospitals, designated H4 (*n* = 2) and H5 (*n* = 2), in Yerevan, Armenia, between January 2019 and April 2019 (Table 1). These isolates were typed as O16:H5-*fimH41* (ARM32 and ARM86) and O25b:H4-*fimH30* (ARM42 and ARM46). All four Armenian E. coli ST131 isolates were resistant to the β-lactam antibiotics ampicillin and amoxicillin-clavulanic acid, the cephalosporin antibiotics ceftazidime and cefepime, and the fluoroquinolone antibiotics norfloxacin and levofloxacin. In addition, two isolates (ARM86 and ARM34) were resistant to the β-lactam antibiotic piperacillin-tazobactam; one isolate (ARM32) possessed intermediate resistance to piperacillin-tazobactam; one isolate (ARM46) was resistant to chloramphenicol; three isolates (ARM32, ARM46, and ARM86) possessed intermediate resistance to the aminoglycoside antibiotic amikacin; and one isolate (ARM46) possessed intermediate resistance to the carbapenem antibiotic imipenem. However, all Armenian E. coli ST131 isolates were sensitive to meropenem (a carbapenem antibiotic).

**TABLE 1:** Antibiotic susceptibility profile of E. coli ST131 isolates recovered in two hospitals in Armenia^*a*^

ID	Sequence type	Date^*b*^	Hospital	Serotype	*fimH*	AMP	PTZ	AMC	CAZ	CPM	Nor	LVX	AMK	IPM	MEM	CHL
ARM32	ST131	07/03/2019	H4	O16:H5	41	R	I	R	R	R	R	R	I	S	S	S
ARM42	ST131	15/04/2019	H5	O25b:H4	30	R	S	R	R	R	R	R	S	S	S	S
ARM46	ST131	11/03/2019	H5	O25b:H4	30	R	R	R	R	R	R	R	I	I	S	R
ARM86	ST131	09/01/2019	H4	O16:H5	41	R	R	R	R	R	R	R	I	S	S	S

a R, resistant; I, intermediate; S, sensitive; AMP, ampicillin (10 mg); PTZ, piperacillin-tazobactam (30/6 mg); AMC, amoxicillin (20 mg)-clavulanic acid (10 mg); CAZ, ceftazidime (10 mg); CPM, cefepime (30 mg); NOR, norfloxacin (10 mg); LVX, levofloxacin (5 mg); AMK, amikacin (30 mg); IMP, imipenem (10 mg); MEM, meropenem (10 mg); CHL, chloramphenicol (30 mg).

b Dates are given in the format day/month/year.

### Phylogenetic analysis of ST131.

**FIG 1:**
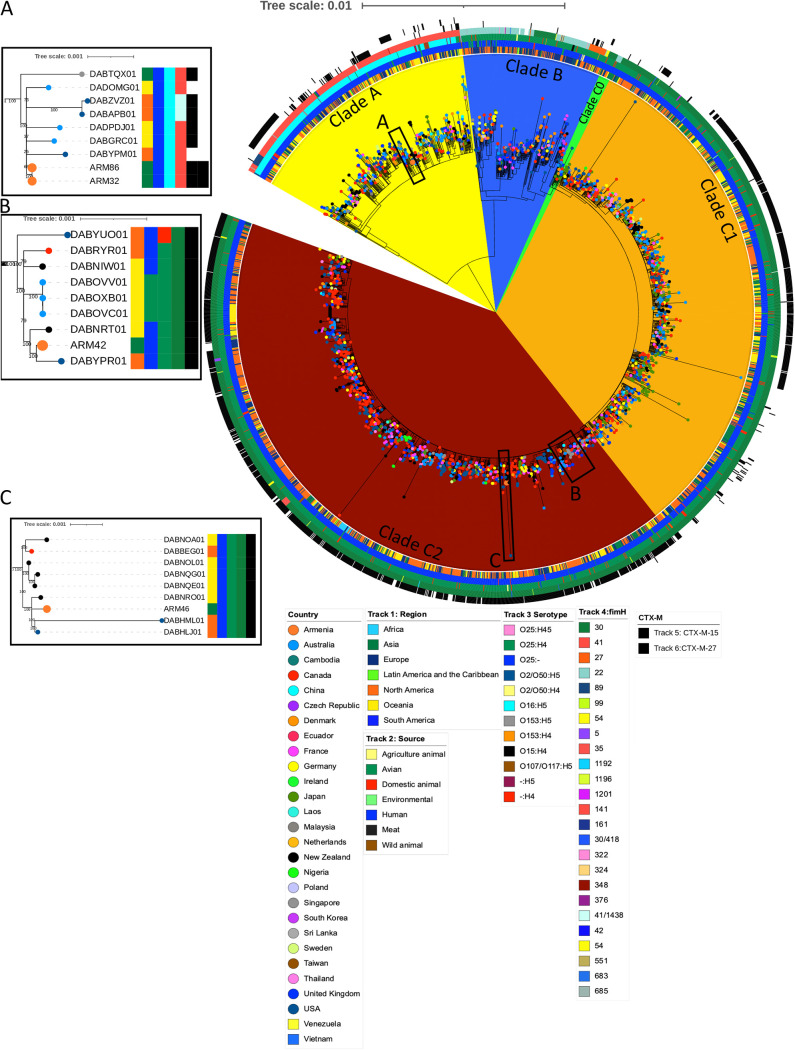
Maximum-likelihood phylogenetic tree of E. coli ST131 showing genetic diversity of the Armenian isolates.

To compare the Armenian E. coli ST131 isolates with those previously reported, a core SNP maximum-likelihood (ML) phylogenetic tree from 11,386 SNP sites with 99 recombination sites filtered out was constructed by aligning the short-read assemblies of our isolates to 2,496 E. coli ST131 isolate draft genomes retrieved from the European Nucleotide Archive (ENA) database that had been recovered from 28 countries (Fig. 1). These isolates were recovered from seven different sources (agriculture animals, avians, domestic animals, the environment, humans, meat, and wild animals), identified as belonging to five clades, 13 serotypes, and 27 *fimH* types (Table 2). Two Armenian *fimH30* isolates (ARM42 and ARM46) were found within clade C2 and were phylogenetically closely related to human isolates recovered from the United States. Specifically, ARM42 was related to an isolate recovered from a blood sample in the United States (DABYPR01), and ARM46 was related to two isolates recovered from human stool samples in New York (DABHLM01 and DABHLJ01), as well as two isolates recovered from humans in New Zealand (ARM42 was related to DABNRT01, and ARM46 was related to DABNRO01, an isolate recovered from a blood sample). All isolates harbored the *bla*_CTX-M-15_ gene. The Armenian *fimH41* isolates (ARM32 and ARM86) were found to belong to clade A and were phylogenetically closely related to human isolates from the United States that were recovered from urine samples in Pittsburgh, PA (DABAPB01), and the Northeast (DABYPM01), as well as human isolates from Australia that were recovered from fecal samples (DADOMG01 and DABGRC01) and a sample of lung fluid (DADPDJ01). Temporal analysis of the E. coli ST131 phylogenetic tree showed no temporal signal within the data set (Fig. S1), with a correlation coefficient of 0.2.

**TABLE 2:** Lists of countries, sources, BAP clusters, and serotypes of ST131 isolates

Country (*n*)	Source (*n*)	Clade (*n*)	Serotype (*n*)	*fimH* allele (*n*)
Armenia (4)	Agriculture animal (3)	A (378)	−:H4 (97)	22 (148)
Australia (294)	Avian (54)	B (221)	−:H5 (9)	27 (26)
Cambodia (3)	Domestic animal (21)	C0 (17)	O107/O117:H5 (5)	30 (1,893)
Canada (581)	Environmental (2)	C1 (826)	O15:H4 (3)	35 (10)
China (10)	Human (2,413)	C2 (1,059)	O153:H4 (3)	41 (337)
Czech Republic (1)	Meat (2)		O153:H5 (3)	42 (1)
Denmark (30)	Wild animal (5)		O16:H5 (346)	54 (10)
Ecuador (10)			O2/O50:H4 (4)	89 (16)
France (118)			O2/O50:H5 (2)	99 (9)
Germany (123)			O25:- (8)	141 (6)
Ireland (1)			O25b:H4 (2,019)	161 (1)
Japan (195)			O25b:H45 (1)	234 (2)
Laos (6)				298 (1)
Malaysia (5)				322 (2)
Netherlands (16)				324 (1)
New Zealand (226)				348 (1)
Nigeria (23)				376 (1)
Poland (1)				551 (1)
Singapore (63)				683 (1)
South Korea (2)				685 (1)
Spain (52)				1192 (1)
Sri Lanka (8)				1196 (1)
Sweden (16)				1201 (2)
Taiwan (4)				22/685 (1)
Thailand (38)				30/418 (3)
United Kingdom (263)				41 (9)
USA (396)				41/1438 (2)
Venezuela (1)				
Vietnam (10)				

**FIG 2:**
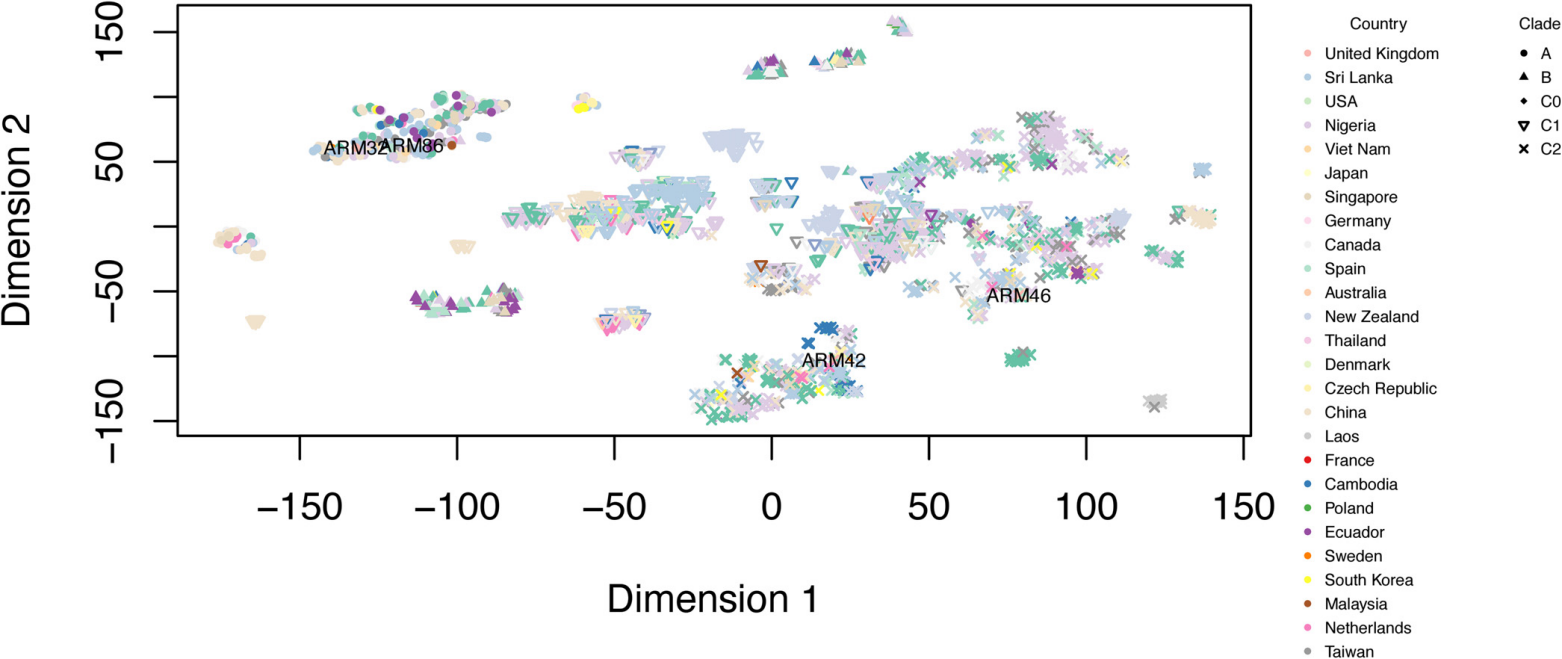
t-SNE plot of accessory genomes of ST131 isolates showing accessory genome differences.

Accessory genome analysis revealed that 46,216 of 49,081 genes shared between 2,500 E. coli ST131 isolates were accessory genes. The t-SNE (*t*-distributed stochastic neighbor embedding) plot revealed that the majority of E. coli ST131 isolates shared many accessory genes with isolates from the same clade, indicating that accessory genes are clonally linked (Fig. 2). We found that isolates belonging to Armenian clade A (ARM32 and ARM86) shared many accessory genes (*r* = 0.93, Z score =1.94, *P* = 0.02) compared to the Armenian clade C2 isolates (ARM42 and ARM46) (*r* = 0.88, Z score = 0.97, *P* = 0.16) (Table S4). ARM42 had a significantly high correlation in its accessory genome (*r* > 0.93, Z score =1.94, *P* < 0.02) with an isolate recovered from a blood sample in Canada (DABRYR01) and an isolate recovered from a human in New Zealand (DABNRT01).

### Virulence gene comparisons.

Overall, we identified 191 virulence genes within 2,500 E. coli ST131 isolates, with the mean number of virulence genes being 87 (range, 50 to 117) (Table S5). Among the Armenian isolates, we found that those (ARM32 and ARM86) belonging to clade A had 104 and 105 virulence genes, respectively, which was significantly higher (*P* < 0.01) than the mean number of virulence genes in clade A (mean, 87; range, 60 to 105). Furthermore, among the Armenian isolates, we also found that two isolates (ARM42 and ARM46) belonging to clade C2 had 88 and 79 virulence genes, respectively, similar to the mean number of virulence genes in clade C2 (mean, 87; range, 51 to 112).

**FIG 3:**
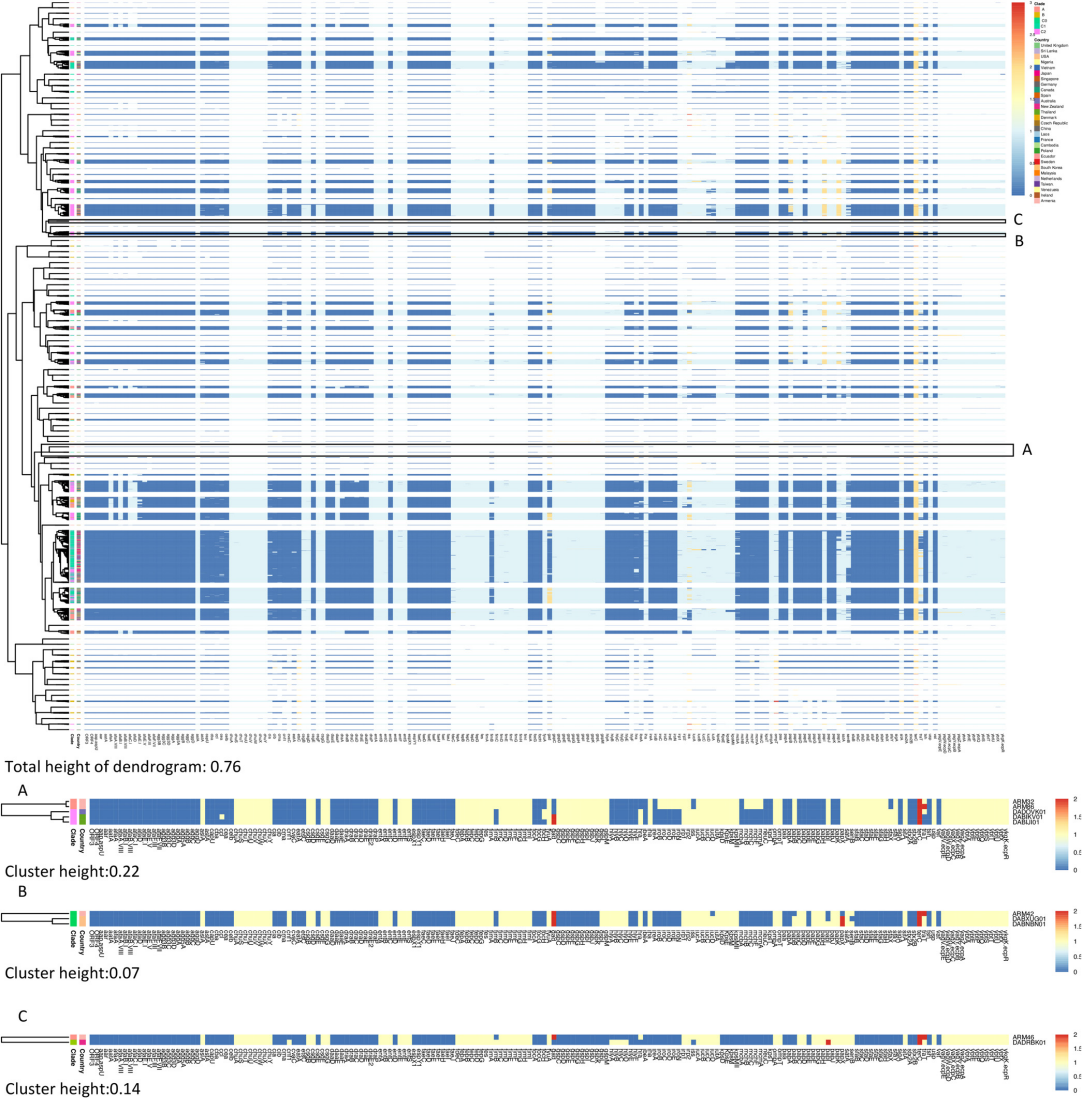
Hierarchy cluster heat map of E. coli ST131 isolates’ virulence-associated gene profiles. (Top) Red, 3 copies of the gene; yellow, 2 copies of the gene; sky blue, 1 copy of the gene; dark blue, absence of gene. (A, B, and C) Red, 2 copies of the gene; yellow, 1 copy of gene; blue, absence of gene.

By constructing the virulence-associated-gene hierarchy cluster heat map, we determined that the Armenian isolates (ARM32 and ARM86) found in clade A clustered with clade C1 isolates DADOVK01 (Jaccard similarity index = 0.87), which was recovered from a rectal swab in Australia, and DABIKV01 (Jaccard similarity index = 0.84) and DABIJI01 (Jaccard similarity index = 0.86), which were recovered from a urine sample in Thailand (Fig. 3A). We found that the microcin H47 operon genes *mchB*, *mchC*, *mchF*, and *mcmA*, the S-fimbrial operon genes *sfaB*, *sfaC*, *sfaD*, *sfaE*, *sfaF*, *sfaG*, *sfaH*, and *sfaY*, the salmochelin operon, *iroBCDE*, and *iroN* were present in clade A Armenian isolates but were absent in other clade A isolates (*n* = 375) in the ENA database. Furthermore, the microcin H47 operon genes were identified in only one other ST131 isolate (DADZMQ01), recovered from Denmark. The S-fimbrial operon and salmochelin operon were present in only 0.2% and 4.3% of the ENA ST131 isolates, respectively. Within clade C2, ARM42 clustered with isolates designated DABNBN01 (Jaccard similarity index = 0.96) and DABXUG01 (Jaccard similarity index = 0.95) (recovered from urine in the United States) (Fig. 3B), whereas ARM46 clustered with a clade A isolate, DADRBK01 (Jaccard similarity index = 0.89) (recovered from urine in New Zealand) (Fig. 3C).

### Antibiotic resistance genotype comparison.

Among 2,500 ST131 isolates, we identified (in total) 350 antibiotic resistance genotypes (genes and mutations), with the mean number of antibiotic resistance genotypes being 65 (range, 52 to 79). Of these, 17 antibiotic resistance genotypes were determined to be a part of the ST131 core resistome (found in 100% of isolates), with a further 57 being found in more than 90% of the isolates (Table S6). The Armenian isolates of clade A (ARM32 and ARM86) possessed 69 and 73 antibiotic resistance genotypes, respectively, which are higher but not significantly higher (*P* = 0.18) than the mean number of antibiotic resistance genotypes in other clade A isolates (mean, 64; range, 55 to 73). The Armenian isolates of clade C2 (ARM42 and ARM46) possessed 69 and 63 antibiotic resistance genotypes, which were similar to the mean number of antibiotic resistance genotypes in clade C2 isolates (mean, 66; range, 56 to 79). Antibiotic resistance genotypes, *dfrA17*, *aadA5*, *qacE*Δ*1*, *sul1*, *bla*_CTX-M-15_, and Escherichia coli *parC* S80I were found to be ubiquitous in Armenian isolates and were not part of the ST131 core resistome (<90% of the isolates) but were still commonly found in ST131 isolates (>40% of the isolates). ARM32, ARM42, and ARM86 also harbored *bla*_OXA-1_, *aac(6′)-Ib-cr4*, *aac(3)-IIe*, *mphA*, and *catB3*. In addition, ARM42 and ARM86 harbored *tetA*; ARM32 and ARM86 harbored *bla*_CTX-M-27_; and ARM86 harbored *APH(3″)-Ib*, *APH(6)-Id*, and *sul2*. The combination of *bla*_CTX-M-15_ and *bla*_CTX-M-27_ found in ARM32 and ARM86 was seen in only one other ST131 isolate (DADOWZ01), a clade C isolate recovered from a blood sample in Australia. The antibiotic resistance phenotypes of three Armenian isolates (ARM42, ARM32, and ARM86) correlated closely with the antibiotic resistance genotypes they harbored. However, we were unable to correlate ARM46 piperacillin-tazobactam, amikacin, imipenem, and chloramphenicol resistance phenotypes to a known antibiotic resistance genotype (Table 3).

**TABLE 3:** Antibiotic resistance genotypes of the Armenian E. coli ST131 isolates that were determined to match their phenotype^*a*^

Isolate	Resistance phenotype	AMR genotype(s)
ARM32	AMP	OXA-1, CTX-M-27, CTX-M-15
	PTZ	OXA-1
	AMC	OXA-1, CTX-M-27, CTX-M-15
	CAZ	CTX-M-27, CTX-M-15
	CPM	OXA-1, CTX-M-27, CTX-M-15
	NOR	*parC* S80I, *gyrA* S83L, *gyrA* D87N
	LVX	*parC* S80I, *gyrA* S83L, *gyrA* D87N
	AMK	*aac(6′)-Ib-cr*

ARM42	AMP	CTX-M-15, OXA-1
	AMC	CTX-M-15, OXA-1
	CAZ	CTX-M-15
	CPM	CTX-M-15
	NOR	*parC* S80I, *gyrA* S83L, *gyrA* D87N
	LVX	*parC* S80I, *gyrA* S83L, *gyrA* D87N

ARM46	AMP	CTX-M-15
	PTZ	NA
	AMC	CTX-M-15
	CAZ	CTX-M-15
	CPM	CTX-M-15
	NOR	*parC* S80I, *gyrA* S83L, *gyrA* D87N
	LVX	*parC* S80I, *gyrA* S83L, *gyrA* D87N
	AMK	NA
	IPM	NA
	CHL	NA

ARM86	AMP	OXA-1, CTX-M-27, CTX-M-15
	PTZ	OXA-1
	AMC	OXA-1, CTX-M-27, CTX-M-15
	CAZ	CTX-M-27, CTX-M-15
	CPM	OXA-1, CTX-M-27, CTX-M-15
	NOR	*parC* S80I, *gyrA* S83L, *gyrA* D87N
	LVX	*parC* S80I, *gyrA* S83L, *gyrA* D87N
	AMK	*aac(6′)-Ib-cr*

a AMP, ampicillin; PTZ, piperacillin-tazobactam; AMC, amoxicillin-clavulanic acid; CAZ, ceftazidime; CPM, cefepime; NOR, norfloxacin; LVX, levofloxacin; AMK, amikacin; IMP, imipenem; MEM, meropenem; CHL, chloramphenicol; NA, not available.

**FIG 4:**
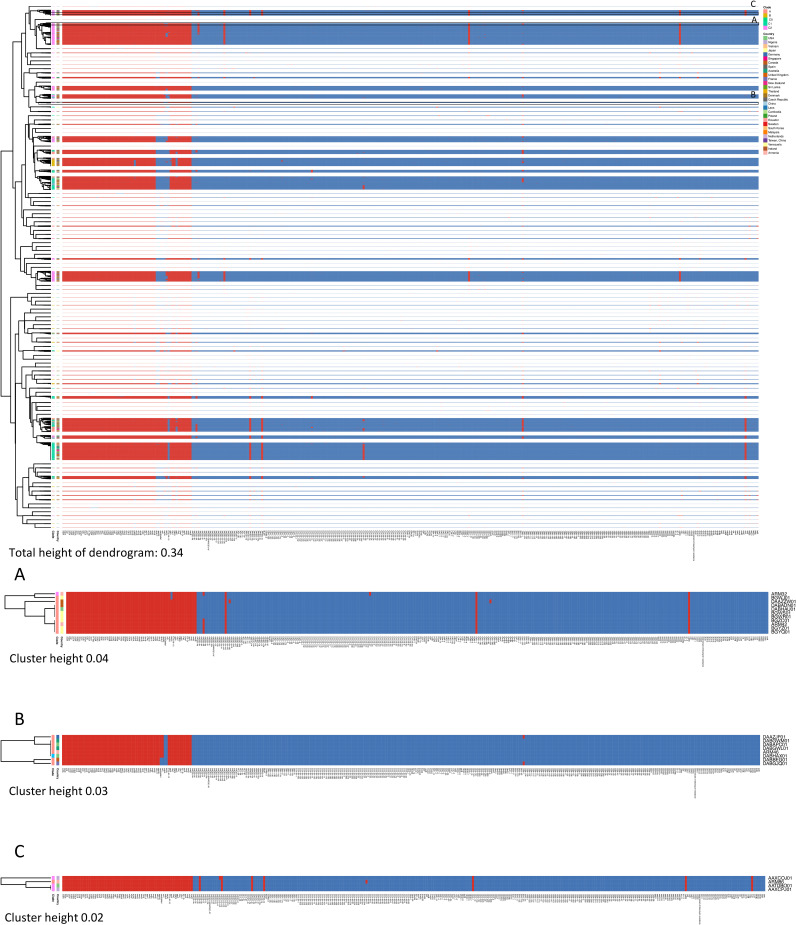
Hierarchy cluster heat map of E. coli ST131 isolates’ antibiotic resistance genotype profiles. Red, presence of gene; blue, absence of gene.

We found that all Armenian isolates clustered based on having antibiotic resistance genotypes similar to those of ENA isolates found in clade C (Fig. 4). Based on their antibiotic resistance genotype, ARM32 and ARM42 (Jaccard similarity index = 0.97) clustered with DABADN01 (Jaccard similarity index = 0.96 and 0.99 for ARM32 and ARM42, respectively) and DAAZZW01 (Jaccard similarity index = 0.93 and 0.96) (recovered in Canada), BGWR01 and BGWS01 (both having a Jaccard similarity index of 0.96 and 0.99 for ARM32 and ARM42, respectively), BGWJ01 (Jaccard similarity index = 0.97), BGZC01, BGYZ01, and BGYQ01 (all having a Jaccard similarity index of 0.97 and 1 for ARM32 and ARM42, respectively) (recovered from Japan), and DABHAU01 (recovered in the United States) and DABFGX01 (recovered in the United Kingdom) (both having a Jaccard similarity index of 0.96 and 0.99 for ARM32 and ARM42, respectively) (Fig. 4A). ARM46 had the same antibiotic resistance genotype as isolates DABHAX01 and DABAPC01 (recovered in the United States) and DABGWM01 and DABGWL01 (recovered in Australia) (Jaccard similarity index = 1) (Fig. 4B). ARM46 also clustered with DAAZJF01 (Jaccard index = 0.98) (recovered in Germany), DABBEG01 (Jaccard similarity index =0.97) (Canada), and DABGJQ01 (Jaccard similarity index = 0.95) (France). Moreover, ARM86 clustered with isolate AAXCOJ01 (Jaccard similarity index = 0.97) and with isolates AAXCPJ01 (recovered in Nigeria) and AATDBO01 (recovered in the United States) (both having a Jaccard index of 0.99) (Fig. 4C).

### Plasmid analysis of the Armenian ST131 isolates.

**FIG 5:**
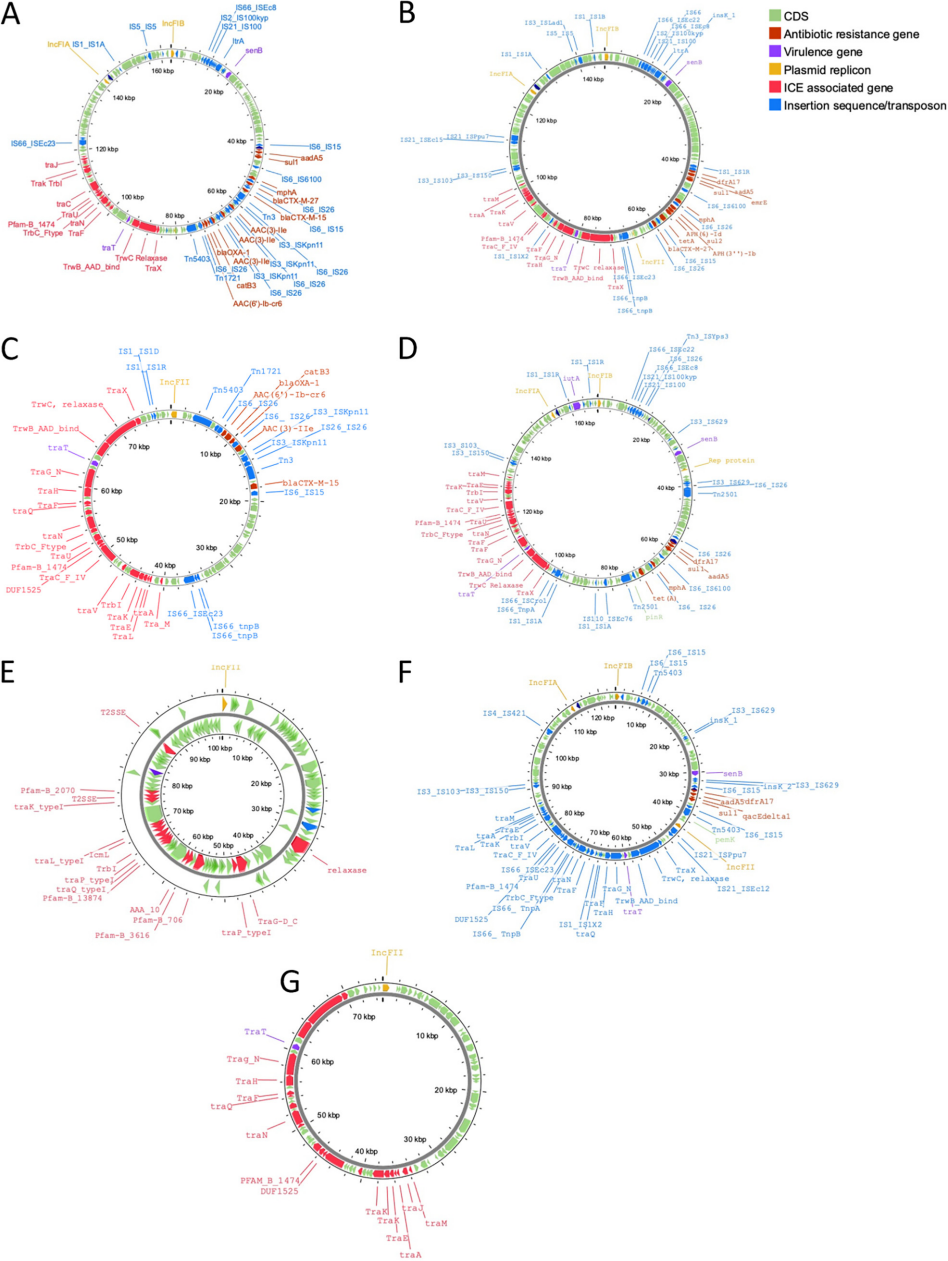
Armenian E. coli ST131 plasmids. (A) ARM32p1 IncF[F2:A2:B20]; (B) ARM86p1 IncF[F1:A2:B20]; (C) ARM86p2 IncF[F2:A−:B−]; (D) ARM42p1 IncF[F36/31:A4:B1]; (E) ARM42p2 IncC/B/O/K/Z; (F) ARM46p1 IncF[F1:A1:B16]; (G) ARM46p1[F2:A−:B−].

Using hybrid genome assembly, we were able to obtain complete assemblies of the Armenian isolates’ plasmids. Two out of four of the Armenian isolates (ARM42 and ARM86) harbored two plasmids, whereas ARM46 harbored three plasmids and ARM32 possessed only a single plasmid. Among the Armenian clade A isolates, the ARM32 plasmid (ARM32p) was typed as an IncF[F2:A2:B20] plasmid (Fig. 5A), ARM86 plasmid 1 (ARM86p1) was typed as an IncF[F1:A2:B20] plasmid (Fig. 5B), and ARM86 plasmid 2 (ARM86p2) was typed as an IncF[F2:A−:B−] plasmid (Fig. 5C). Among the Armenian clade C2 isolates, ARM42 plasmid 1 (ARM42p1) was identified as an IncF[F31/36:A4:B1] plasmid (Fig. 5D), ARM42 plasmid 2 (ARM42p2) as an IncB/O/K/Z plasmid (Fig. 5E), ARM46 plasmid 1 (ARM46p1) as an IncF[F1:A1:B16] plasmid (Fig. 5F), ARM46 plasmid 2 (ARM46p2) as an IncF[F2:A-:B-] plasmid (Fig. 5G), and ARM46 plasmid 3 as a Col(MG828) plasmid which possessed only a plasmid replicon and a single hypothetical gene.

**FIG 6:**
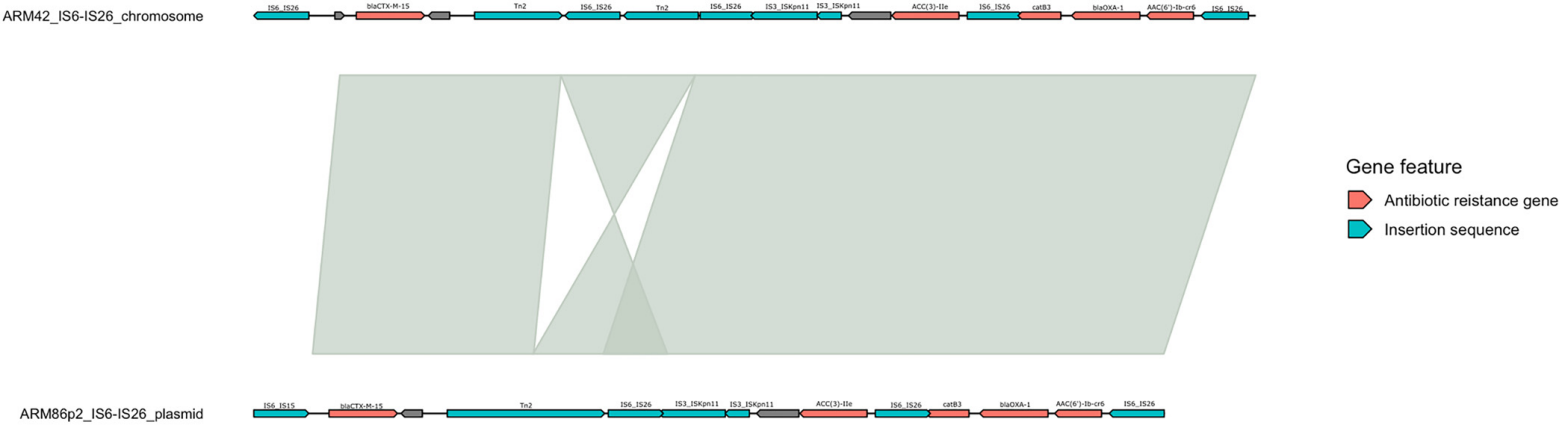
E. coli CTX-M-15 IS*26* antibiotic resistance islands in ARM42 and ARM86 showing high genetic similarities.

Five of the plasmids harbored antibiotic resistance genes within an IS*26* genomic island, with ARM32p (*n* = 13) harboring the most antibiotic resistance genes, followed by ARM86p1 (*n* = 10), ARM86p1 (*n* = 10), ARM86p2 (*n* = 5), and ARM46p1 (*n* = 4). The ESBL gene *bla*_CTX-M-15_ was present in the Armenian clade A isolate plasmids ARM32p and ARM86p2 but was chromosomal in clade C2 Armenian isolates. The *bla*_CTX-M-15_ gene in ARM46 was integrated into the chromosome via an IS*Ecp1-*CTX-M-15 transposition unit, whereas in ARM42, the *bla*_CTX-M-15_ gene was integrated within the chromosome as part of the IS*26* genomic island containing *aac(3)-IIe*, *catB3*, *bla*_OXA-1_, and *aac(6′)-Ib-cr6* antibiotic resistance genes. Furthermore, the *bla*_CTX-M-15_ IS*26* genomic island in ARM42 had a high degree of genetic synteny with the *bla*_CTX-M-15_-IS*26* genomic island found in ARM86p2 (Fig. 6) and high genetic similarities within the chromosome in complete E. coli ST131 genomes obtained from the National Center for Biotechnology Information (NCBI) nucleotide repository (Table S7).

**FIG 7:**
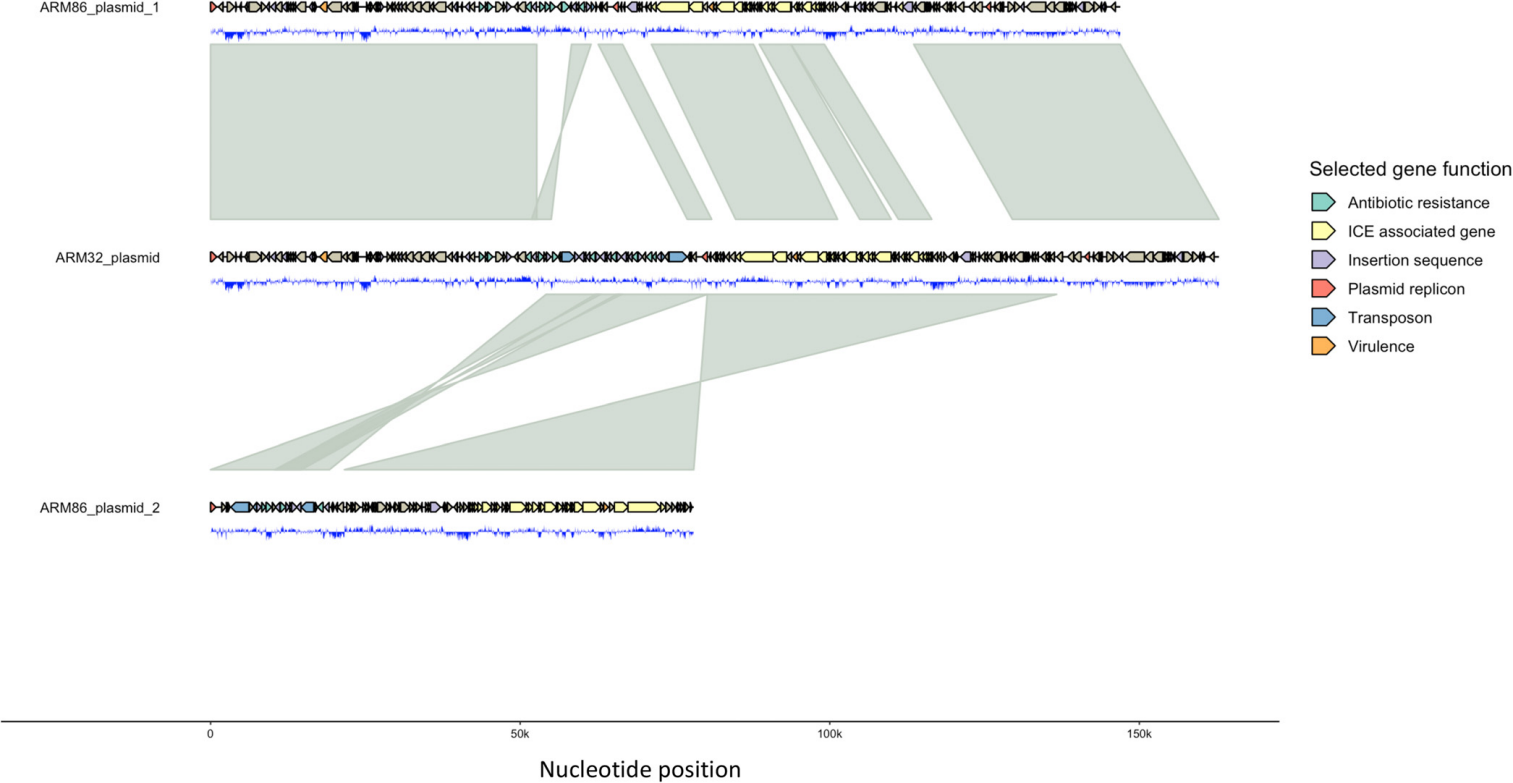
Plasmid symmetry comparison of ARM32 and ARM86 showing two separate recombination cointegration events from ARM86 plasmid 2 into the ARM86 plasmid backbone to form the ARM32 plasmid.

Synteny analysis of the Armenian isolates’ plasmids revealed that ARM32p had high genetic similarities with both ARM86p1 and ARM86p2 (Fig. 7). Further analysis of ARM32p identified two regions of reverse synteny with ARM86p2 adjacent to each other and flanked by genomic sequences that had synteny to ARM86p1. These regions of synteny consisted of ARM86p2 between its IncFII replicon and IS*15* and contained the ARM86p2 antibiotic resistance genes *bla*_CTX-M-15_, *aac(3)-IIe*, *cat3B*, *bla*_OXA-1_, and *aac(6′)-Ib-cr6*. The other region of synteny with ARM86p2 contained the integrating conjugative element (ICE)-associated genes. There were multiple regions of synteny to ARM86p1 within the ends and middle sections of the same ICE-associated gene region. There was also a change in sequence orientation of the ARM86p IS*26*-*bla*_CTX-M-27_-IS*903B*-IS*15* region in ARM32p. ARM32p had all the antibiotic genes found in ARM86p2 but had only 6 of the 10 antibiotic resistance genes found in ARM86p1; it was missing *tetA*, *APH(6)-Id*, *APH(3′)-Ib*, and *sul2*. Moreover, ARM32p possessed 3 copies in direct repeat of a genetic region found in ARM86p1 consisting of IS*26*, IS*Kpn11*, the AAA family ATPase gene, *aac(3)*, *IIe*, and IS*26*.

Our data analysis showed high genetic similarities of E. coli ST131 draft genomes obtained from the ENA database to the Armenian isolates’ plasmids. We found that ARM42p1 had a high score (>0.999) of genetic identity to clade C2 ENA isolates that harbored the IncF[F31/36:A4:B1] genotype and had the same antimicrobial resistance (AMR) as ENA isolates recovered from Japan, Canada, the United Kingdom, New Zealand, Australia, and the United States. The Japanese isolate BGZA01 had the highest genetic similarity contained in its genome, whereas the Canadian isolate DABABP01 (recovered from a blood sample) had the highest BLAST coverage (0.93) relative to ARM42p1. ARM46p1 had a high score (>0.999) of genetic identity to clade C2 ENA isolates that possessed the IncF[F1:A1:B16] genotype and had the same AMR genes as ENA isolates recovered from France, Canada, the United Kingdom, New Zealand, Australia, and the United States. The U.S. isolate AATDBO01 (recovered from a blood sample) had the highest genetic identity score and the Australian isolate DADPEZ01 had the highest BLAST coverage (0.92) relative to ARM46p1. For the other Armenian isolate plasmids, we did not find genetic identities higher than >0.999 contained within the ENA isolates. The ENA isolate DADOWZ01, which had both the *bla*_CTX-M-15_ and *bla*_CTX-M-27_ genes, had the same IncF type as ARM32 but was not genetically close to ARM32p (identity score, 0.983; BLAST coverage, 0.64), ARM86p1 (identity score, 0.989; BLAST coverage, 0.47), and ARM86p2 (identity score, 0.962; BLAST coverage, 0.75).

## DISCUSSION

In this study, we report for the first time the antibiotic resistance profiles and genetic features of four UPEC ST131 isolates recovered from patients in Armenia, where genomic surveillance is lacking. By combining the short-read sequencing for high base calling accuracy to infer Armenian isolates’ genotype and phylogeny and long-read sequencing to resolve repetitive elements and identify structures and positioning of MGE within the chromosome and plasmid, we were able to perform an in-depth genomic analysis of the Armenian E. coli ST131 isolates (20). We found that two Armenian ST131 isolates were genotyped as *H*30Rx/clade C2 (ARM42 and ARM46), one of the most prevalent E. coli ST131 genotypes that cause UTI globally (2, 21). The other two isolates were genotyped as *H*41/clade A (ARM32 and ARM86), which has recently emerged as one of the most common ST131 genotypes causing UTI in Australia, New Zealand, and China (15, 22, 23). Intriguingly, ARM32 and ARM86 are phylogenetically closely related to each other and share many of the same accessory genes but were recovered from patients in two different hospitals, which may indicate a wide community transmission of this strain. Furthermore, we found that the Armenian isolates were phylogenetically more closely related to isolates recovered from Australia, New Zealand, and the United States and were more likely to share accessory genes such as those for virulence factors and antibiotic resistance genotypes than isolates recovered from other countries. Moreover, using long-read sequencing, we were able to determine that genetic elements in the ENA isolates (those that were recovered from countries such as Australia, New Zealand, and the United States) had high genetic similarities with the Armenian isolates. We hypothesize that because there are large Armenian diasporas in Australia, New Zealand, and especially the United States, with close family links in Armenia, it seems plausible that the transmission could have occurred through members of these Armenian communities (24, 25).

The antibiotic resistance phenotypes of Armenian ST131 isolates were consistent with that of ST131 isolates except in the case of ARM46, which demonstrated intermediate resistance to the carbapenem antibiotic imipenem (26, 27). Antibiotic resistance genotyping also showed that carbapenem and chloramphenicol resistance was absent in ARM46, potentially indicating that it may have developed resistance via an unknown mechanism. Moreover, we were unable to determine the antibiotic resistance genotype for amikacin and piperacillin-tazobactam in ARM46, which, however, was determined for other Armenian isolates due to the identification of associated *aac(6′)-Ib-cr* and *bla*_OXA-1_ genes. For all isolates, ESBL production was linked to the *bla*_CTX-M-15_ gene, which was plasmid borne in Armenian *H*41 isolates and chromosomal in *H*30Rx isolates. Chromosomal *bla*_CTX-M-15_ is generally considered rare in E. coli ST131 isolates, as IncF *bla*_CTX-M-15_ plasmids are very stable within the population even in the absence of antibiotic pressure (28–30). However, the general prevalence of chromosomal *bla*_CTX-M-15_ is still relatively unknown in whole-genome sequencing studies due to the use of short-read sequencing platforms, which have difficulties in differentiating between plasmid-borne and chromosomal genetic features in comparison to long-read sequencing (17, 31). The chromosomal integration of *bla*_CTX-M-15_ in ARM46 was related to IS*Ecp1-bla*_CTX-M-15_, which previously was identified as chromosomal in ST131 isolates recovered in Zambia and Ireland (30, 32), whereas the chromosomal integration of *bla*_CTX-M-15_ in ARM42 was found within an IS*26* antibiotic resistance genomic island which was also present in ST131 isolates’ chromosomes (obtained from the NCBI nucleotide database). Moreover, this IS*26* antibiotic resistance island was also present in the Armenian *H*41 isolates’ plasmids, which may suggest that this resistance genomic island was common among the Armenian ST131 isolates.

Moreover, we were able to determine that the Armenian *H*41 isolates harbored both *bla*_CTX-M-15_ and *bla*_CTX-M-27_ genes. Intriguingly, we found that the ARM32 IncF[F2:A2:B20] plasmid harboring both *bla*_CTX-M-15_ and *bla*_CTX-M-27_ is a fusion of ARM86 *bla*_CTX-M-15_ on the IncF[F2:A−:B−] plasmid and *bla*_CTX-M-27_ on the IncF[F1:A2:B20] plasmid, most likely from a recent recombination event between the plasmids’ IS*26* antibiotic genomic islands (33, 34). Recombination events between IncF plasmids have been reported; however, the movement of the *bla*_CTX-M-15_ gene into the *bla*_CTX-M-27_ plasmid backbone in the E. coli ST131 genetic background is undocumented (13, 35, 36). The fusion plasmid of ARM32 also harbored many of the antibiotic resistance genes on the two plasmids found in ARM86, which may make the horizontal transfer of multiple antibiotic resistance genes easier. Furthermore, we found an increased copy number, detected by long-read sequencing with the ability to resolve repetitive elements, of the aminoglycoside antibiotic resistance genes *aac(3)-IIe* (1 copy in ARM86 and 3 copies in ARM32), which has been shown to increase the MIC of tobramycin (37–39). The evolution observed between these ARM32 and ARM86 plasmids demonstrates that recombination of *bla*_CTX-M-15_ can occur in the *bla*_CTX-M-27_ plasmid backbone within ST131-*fimH41* backgrounds, which has not been seen before in other ST131 lineages, as they are restricted to carrying certain IncF plasmid types (13). Only one ENA isolate (DADOWZ01) that harbored both *bla*_CTX-M-15_ and *bla*_CTX-M-27_ genes possessed the IncF[F2:A2:B20] type, which suggests that this plasmid genotype may be the only genotype that can harbor both *bla*_CTX-M-15_ and *bla*_CTX-M-27_. Moreover, as antibiotic resistance genes within IncF plasmids are acquired via homology recombination with common features in the IncF plasmid rather than via transposition, the development of the IncF[F2:A2:B20] genotype is most likely to occur due to allele exchange of two different IncF plasmids’ IncFII replicons (34, 36, 40).

The Armenian *H*41 isolates harbored more virulence genes than ENA isolates that belonged to the same genotype. In particular, the Armenian isolates harbored a genomic island which contained the S-fimbrial operon (encoding adhesin factor), the salmochelin operon (encoding an iron siderophore), and a microcin H47 operon (which encodes a bacteriocin that inhibits other enteric pathogens) (41); this genomic island is known but uncommon in ST131 isolates. Previous reports of isolates harboring the microcin H47–salmochelin–S-fimbrial genomic island generally showed increased virulence, suggesting that these genes provide a fitness advantage for E. coli, contributing to its survival and acquisition of virulence factors (42, 43). Moreover, salmochelin and microcin H47 genes may have a role in promoting urinary tract colonization, as observed by the upregulation of salmochelin and microcin H47 genes when isolates are grown in urine (42–45). As ST131 isolates are considered the most common sequence type causing UTI, the lack of this pathogenicity island suggests that these virulence factors may not be entirely necessary for them to be successful in causing UTI. Moreover, the mechanism of the success of ST131 in causing UTI compared to other E. coli sequence types is still relatively unknown (46–49).

In summary, we report for the first time genetic features and transmission of ESBL-producing E. coli ST131 isolates recovered in Armenia using both short- and long-read sequencing technologies to provide in-depth genomic analysis to better understand Armenian isolates’ genotype, phylogeny, and MGE structure and position within the chromosome and plasmid. Although we had only 4 isolates belonging to two different ST131 genotypes, we were able to identify the close relatedness of these isolates to those recovered in the United State, Australia, and New Zealand. Our data show that Armenian isolates included in this study possessed genetic features not commonly identified in other E. coli ST131 isolates, including the *bla*_CTX-M-15_ gene integrated into the ST131-*H*30Rx isolates’ chromosomes and the acquisition of the pathogenicity island containing the S fimbrial, salmochelin siderophore, and microcin H47 virulence genes in ST131-*H*41 isolates. Moreover, using hybrid assembly of short- and long-read sequencing in ST131-*H*41 isolates, we detected the formation of IncF[F2:A2:B20] containing both *bla*_CTX-M-15_ and *bla*_CTX-M-27_ derived from the recombination of genes from an IncF[F2:A−:B−] *bla*_CTX-M-15_-associated plasmid into an IncF[F1:A2:B20] *bla*_CTX-M-27_-associated plasmid backbone. Further genomic surveillance of ESBL E. coli ST131 isolates in Armenia is necessary to determine whether the genetic features identified in our study are widely disseminated in other E. coli ST131 UTI isolates, which could contribute to global AMR transmission.

## MATERIALS AND METHODS

### Bacterial isolation and identification.

Twelve ESBL-producing UPEC isolates were received from medical microbiology laboratories of five hospitals in Armenia between January 2019 and August 2019 (Table S1) (50). All isolates were recovered from urine specimens from hospitalized patients. The isolates were identified as E. coli using matrix-assisted laser desorption ionization–time-of-flight mass spectroscopy (MALDI-TOF MS) as described previously (51). Four of 12 isolates were identified as E. coli ST131 by multilocus sequence typing (MLST) from whole-genome sequencing using mlst (https://github.com/tseemann/mlst [accessed July 2021]) and were selected for use in this study.

### Antibiotic susceptibility testing.

The identification and susceptibility profiles of these four and other *E.coli* isolates were reported previously (50); in brief, each isolate was tested against a panel of 10 antibiotics using the disk diffusion method. The antibiotics included were ampicillin (10 mg), piperacillin-tazobactam (30/6 mg), amoxicillin-clavulanic acid (20/10 mg), ceftazidime (10 mg), cefepime (30 mg), norfloxacin (10 mg), levofloxacin (5 mg), amikacin (30 mg), imipenem (10 mg), meropenem (10 mg), and chloramphenicol (30 mg) (Mast Group, Merseyside, UK) according to the European Committee on Antimicrobial Susceptibility Testing (EUCAST) protocol (52). Isolates were determined to be ESBL producing if they showed resistance to ceftazidime and cefepime.

### Genome sequencing and assembly.

The 12 E. coli isolates were whole-genome sequenced using the Illumina HiSeq platform. Genomic DNA was extracted using the TIANamp bacterial DNA kit (Tiangen, China), and paired-end sequencing libraries were constructed using Nextera XT DNA sample preparation kits or TruSeq DNA HT sample preparation kits (Illumina, USA) following the manufacturer’s instructions.

The quality of short reads was checked using FastQC, with low-quality reads trimmed by Trimmomatic (53). Trimmed reads were *de novo* assembled using SPAdes (54).

Four of 12 isolates that were identified as E. coli ST131 were further sequenced for detailed chromosomal and plasmid genetic investigation using the Oxford Nanopore Technologies MinIon 1B sequencing platform. Genomic DNA for long-read sequencing was extracted using a Qiagen MagAttract HMW DNA kit (Qiagen, Germany), and the long-read library was constructed using a ligation sequencing kit (SQK-LSK109) (Oxford Nanopore Technologies, UK) and run on an R9.4.1 flow cell (Oxford Nanopore Technologies, UK). Guppy v6.3.4 was used for long-read base calling, filtering out reads with a Q score of <9 (55).

Long and short reads were combined for hybrid genome assembly using two different assembly pipelines depending on the sequencing depth of long reads. All isolates were assembled using the Unicycler assembly pipeline for the detection of plasmids less than <10 kb (56, 57). The genome of one isolate (ARM42) was assembled using only Unicycler. Isolates ARM32, ARM46, and ARM86 (which had a long-read sequence depth greater than 50×) were also assembled using Canu, Flye, Miniasm+Minipolish, Raven, NECAT, and NextDenovo/NextPolish (58–63). A consensus of the long-read assemblies was constructed via Trycycler (64). The final Trycycler assemblies were polished with the short-read sequences of the same isolates using Polypolish and POLCA (65, 66).

### Genome selection for phylogenetic and genomic comparison.

A total of 2,496 draft E. coli ST131 genomes obtained from the ENA database were selected for genomic comparison (accessed January 2022) (Table S3). The isolates were selected based on the criteria that they (i) belonged to ST131 as determined with the mlst (https://github.com/tseemann/mlst accessed July 2021) typing scheme (PubMLST) (accessed January 2022) and (ii) were accompanied by data on their isolation, including date, source, and country.

### Phylogenetic tree analysis.

To reconstruct the core SNP maximum-likelihood (ML) phylogenetic tree, the short sequence reads of E. coli ST131 isolates recovered from Armenia and the isolates from the ENA database were first aligned with the ST131 reference genome EC958 (accession no. HG941718.1) using Snippy (https://github.com/tseemann/snippy). Recombination within the genome was filtered out using Gubbins (67). A phylogenetic tree was constructed using IQ-TREE v2.1.2 with the best model selected by ModelFinder and with ultrafast bootstrap replication set to 1,000 (68–70). The phylogenetic tree was visualized and annotated using iTOL (71). The temporal signal of the phylogenetic tree was determined by TempEst (72).

### Genome annotation.

E. coli ST131 isolate genomes were annotated using Prokka (73). The serotype was identified using ECtyper (74). Virulence-associated genes for each isolate were screened using ABRicate software (https://github.com/tseemann/ABRicate) in conjunction with the VirulenceFinder and VFDB database combined for virulence detection. Antibiotic resistance genotype was identified using Resistance Gene Identifier (RGI) software in conjugation with the Comprehensive Antibiotic Resistance Database (CARD) (accessed January 2022) (75). Antibiotic resistance related to deletion of outer membrane protein genes was also detected (76, 77). A hierarchy cluster heat map of isolated antibiotic resistance genotypes and virulence-associated genes was constructed using the R package Pheatmap. The *fimH* allele type, presence of the *ybbW* G723A SNP in *H*30Rx isolates, and IncF replicon sequence type (RST) were determined using SKA (https://github.com/simonrharris/SKA) in conjunction with the FimTyper and pMLST IncF RST database (accessed August 2022) (78–80).

For Armenian E. coli ST131 isolates, plasmids found in the hybrid genome assembly were typed using MOB-suite and pMLST (81, 82). ABRicate was used to screen for plasmid replicon and insertion sequences (IS) using the PlasmidFinder and ISfinder database (83, 84). ICE-associated genes were screened using ICEfinder (85). Plasmid structure was visualized using Proksee (https://proksee.ca/).

### Pangenome analysis.

The E. coli ST131 pangenome was constructed using both Armenian and ENA isolates combined using Roary with the BLASTp percentage identity cutoff set at 90% (86). A t-SNE plot of the accessory gene was plotted using the R package Rtsne (https://github.com/jkrijthe/Rtsne) and the pairwise Pearson correlation using the R package Hmisc (https://cran.r-project.org/web/packages/Hmisc/index.html).

### Genetic synteny and plasmid similarity analyses.

Plasmid synteny between Armenian isolates was identified from the hybrid genome assembly using minimap2 and visualized using the R package gggenomes (https://github.com/thackl/gggenomes) (87). Mash Screen was used to determine whether the E. coli ST131 isolates’ draft genomes contained sequences matching those in the hybrid assembly of the Armenian isolates’ plasmids, with only those with a Mash Screen identity of >0.995 reported (88). Pyani was used to measure BLAST coverage of the sequence in the ENA database to the Armenian isolates’ plasmids (89).

### Statistical analysis.

Student’s *t* test was used to determine the significant difference between the number of virulence and antibiotic resistance genotypes between isolates. Z-scored normalization was used to determine the significance between accessory gene correlation data. A Jaccard similarity index was used to determine the similarity coefficient between two isolates’ virulence genes and antibiotic resistance gene profiles.

### Data availability.

The short- and long-read sequencing data generated in this study were deposited in the ENA database under the accession numbers ERS14467679 (ARM32), ERS14467680 (ARM42), ERS14467681 (ARM46), and ERS14467682 (ARM86). Individual accession numbers for the genome sequencing data are included in Table S2. Genome assemblies of the isolates are included in Table S2 and deposited in the ENA database under the accession numbers GCA_951802855 (ARM32), GCA_955652485 (ARM42), GCA_951803545 (ARM46), and GCA_951802865 (ARM86). Both Illumina short reads and Oxford Nanopore long reads were uploaded to the ENA repository (project PRJEB51925) (Table S2).
