# Supplementary material for: Short- and Long-Read Sequencing Reveals the Presence and Evolution of an IncF Plasmid Harboring blaCTX-M-15 and blaCTX-M-27 Genes in Escherichia coli ST131
Source: Microbiol Spectr. 2023 Jul 19;11(4):e00356-23. doi: 10.1128/spectrum.00356-23 (PMC10433869; doi:10.1128/spectrum.00356-23)
Supplement: Supplemental file 1 — Supplemental material. Download spectrum.00356-23-s0001.pdf, PDF file, 0.6 MB [file spectrum.00356-23-s0001.pdf]

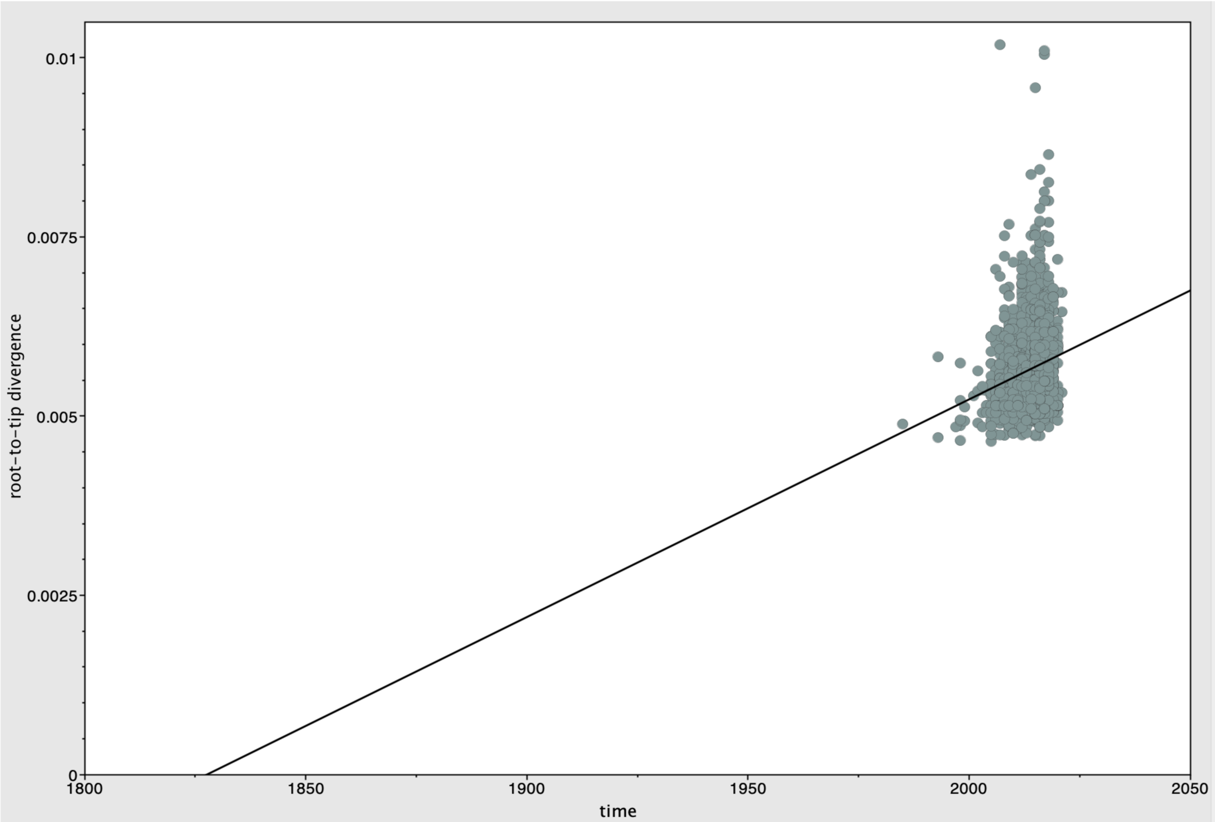

Figure S1:Root-to-tip regression analyses of ST131 isolates showing no temporal signal within the dataset.
